# The Associations of Common Genetic Susceptibility Variants with Breast Cancer in Jordanian Arabs: A Case-Control Study

**DOI:** 10.31557/APJCP.2020.21.10.3045

**Published:** 2020-10

**Authors:** Laith N AL-Eitan, Doaa M ababa’h, Hatem A Aman

**Affiliations:** 1 *Department of Applied Biological Sciences, Jordan University of Science and Technology, Irbid 22110, Jordan. *; 2 *Department of Biotechnology and Genetic Engineering, Jordan University of Science and Technology, Irbid 22110, Jordan.*

**Keywords:** Breast cancer, CASP8, FGFR2, IGF1, LSP1, MAP3K1, Jordanian

## Abstract

Objective: In Jordan, breast cancer (BC) affects a substantial proportion of Jordanian women, highlighting the need for studies to be carried out regarding the genetic component of the disease. The aim of the present study was to investigate the interaction between BC risk and prognosis and polymorphisms in genes (*ATM, CASP8, FGFR2, FN1, IGF1, LSP1, MAP3K, MMP7,* and *RHOC*) that were chosen for this study previously reported as having a role in the disease. Materials and Methods: Blood samples were collected from 242 BC patients and 231 disease-free volunteers recruited from the Jordanian population. DNA was extracted from blood and each sample was sent to the Australian Genome Research Facility for genotyping. Results: The *rs1219648 SNP* of the *FGFR2* gene was the only investigated variant to show any direct association with BC in Jordanian women (p-value = 0.04). However, the CASP8rs6760993 SNP was found to be significantly associated with BC (p-value = 0.04) when using the dominant model. Other gene polymorphisms showed varying levels of association between some investigated SNPs and different BC risk and prognostic factors. Conclusion: Despite reports to the contrary in other populations, most of the investigated genes and their respective SNPs did not show any significant association with BC in Jordanian women. Our results underline the need for independent BC research to be carried out in the Jordanian population to decipher the genetic basis of the disease.

## Introduction

Breast cancer (BC) is the most common malignancy in Jordanian women, accounting for 37.3% of female cancers reported in that country (Abdel-Razeq et al., 2015; AL-Eitan et al., 2017; AL-Eitan et al., 2019). Although its etiology is still not clearly understood, BC risk and development is known to be influenced by a combination of endogenous hormone levels, lifestyle factors, and individual genetic susceptibility (Hankinson et al., 2004; AL-Eitan et al., 2019; AL-Eitan et al., 2020). Around 5-10% of BC cases are caused primarily by genetic mutations (AL-Eitan and Rababa’h, 2019), and of those, the vast majority of genetic influence lies with the *BRCA1* and *BRCA2 *mutations (Gage et al., 2012; AL-Eitan et al., 2017). The remainder of genetic susceptibility to BC can be attributed to mutations that lead to the activation and disruption of proto-oncogenes and tumor suppressor genes, respectively (Lee and Muller, 2010; AL-Eitan et al., 2017; AL-Eitan et al., 2019). Proto-oncogenes are those genes that are involved in cell growth and proliferation, with potential examples including the *ATM, FGFR2, FN1, IGF1, MAP3K, MMP7,* and *RHOC* genes, while the *CASP8* and* LSP1* genes have been reported to possess tumor-suppressive properties in certain types of cancer.

Serine/threonine kinase expression has been found to be frequently modulated in human cancers (Capra et al., 2006). The *ATM serine/threonine kinase (ATM)* gene is involved in the activation of the DNA damage checkpoint, and mutations in this gene are responsible for the autosomal recessive syndrome ataxia-telangiectasia (Lavin et al., 2006). In addition, studies have shown that unaffected carriers of ATM mutations are more susceptible to BC compared to controls and have a lifetime BC risk of more than 25% (Renwick et al., 2006; Ahmed and Rahman, 2006). Similarly, the *mitogen-activated protein kinase 1 (MAP3K1)* gene is a serine/threonine kinase that activates mutagenic downstream signaling pathways, and its mutations have been significantly associated with luminal BC (Jerzak et al., 2018). In fact, MAP3K1 mutations resulted in increased susceptibility to BC and were especially associated with estrogen- and progesterone-receptor positive BC tumors (Pham et al., 2013).

Growth factors and their receptors have also been implicated in cancer development, particularly in tumor metastasis (Rebbeck et al., 2008). The fibroblast growth factor receptor 2 *(FGFR2) *genes are involved in tissue repair, and its mutated forms are associated with autosomal dominant skeletal and cranial disorders (Yang et al., 2011). Moreover, *FGFR2* mutations have been reported in a diverse range of cancer types including BC, and FGFR inhibitors reported to have a direct anti-tumor effect on cancer cells (Jang et al., 2000; Helsten et al., 2016). In a similar fashion, the insulin-like *growth factor 1 (IGF1) *gene exerts anabolic effects via suppression of apoptosis, and high expression levels of this gene play an important role in cancer (Katoh, 2016). Furthermore, a large number of studies point towards the role of the IGF1 system in BC initiation and development (Brahmkhatri et al., 2015).

Aberrant expressions of various membrane-associated G-proteins, matrix metalloproteinase, and glycoproteins have been extensively recorded in tumors (Christopoulos et al., 2015; Bar-Shavit et al., 2016). The *RHOC* gene encodes for the RhoC signaling G protein that, when overexpressed, is involved in cell proliferation and tumor malignancy (Gialeli et al., 2011). In the context of BC, RhoC overexpression is associated with a worse prognosis and is needed for tissue invasion to occur (Horiuchi et al., 2003).

Likewise, the *matrix metalloproteinase-7 (MMP7) *gene, which is normally responsible for tissue remodeling, is overexpressed in cases of tissue invasion as well as tumor formation (Lang et al., 2017). Different polymorphisms in the *MMP7* gene have been associated with both improved and worsened BC prognosis in Chinese patients (Basu et al., 2015). Finally, the *fibronectin 1 (FN1)* gene is an extracellular matrix glycoprotein that is involved in cell proliferation under homeostatic conditions but promotes metastasis of tumor cells when its expression is modulated (Beeghly-Fadiel et al., 2009). Moreover, it has been reported that FN1 expression levels are associated with BC prognosis and invasion (Wang and Hielscher, 2017; Wang et al., 2018). On the other hand, high expression of the caspase 8 (CASP8) and lymphocyte-specific protein 1* (LSP1)* genes are associated with tumor inhibition in most cases (Helleman et al., 2008; Zhang et al., 2016). CASP8 normally functions as a part of cell apoptosis, and *CASP8 *mutations have been associated with resistance to apoptosis as well as malignant transformation in head and neck cancer (Graf et al., 2014; Li et al., 2014).

Furthermore, CASP8 mutations exacerbate the risk of certain BC subtypes, but they are not associated with adverse BC survival rates (Ando et al., 2013; Park et al., 2016). Similarly, the *LSP1* gene has been reported to inhibit hepatocellular carcinoma growth as a part of its normal function, while LSP1 mutations have been found to increase BC susceptibility in both Caucasian and Han Chinese women (Zhang et al., 2016; Pu et al., 2017; Vachon et al., 2012). Since lifestyle factors and endogenous hormone levels also influence BC risk and development, the main aim of this study is to ascertain whether there is an association between BC risk and prognosis and single nucleotide polymorphisms (SNPs) in certain genes in the Jordanian population. The *ATM, CASP8, FGFR2, FN1, IGF1, LSP1, MAP3K, MMP7*, and *RHOC* genes were chosen for this study because of their reported roles in BC in populations other than Jordanians such as Caucasian (Chen et al., 2015; Easton et al., 2007; Zhang et al., 2011; Sadek et al., 2017).

## Materials and Methods

*Subjects*

**Figure 1:**
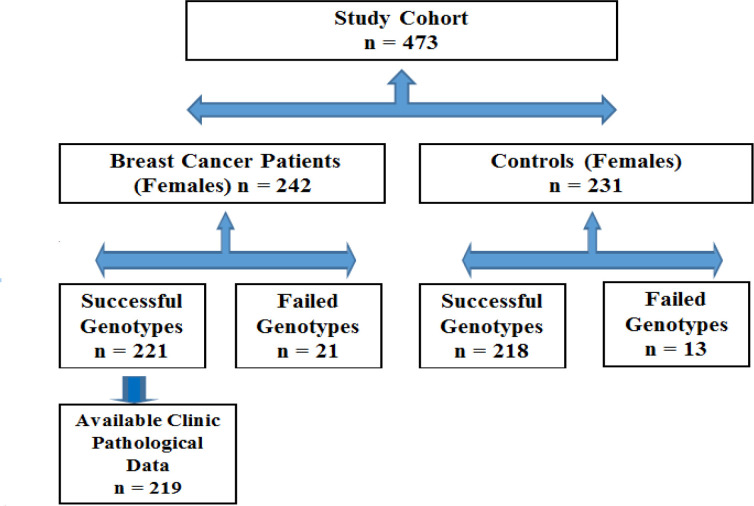
A Consort Diagram Of BC Patients and Controls Detailing Inclusion and Exclusion Criteria

This study conducted on 221 BC female patients and 218 healthy individuals recruited from the Jordanian Arab population as shown in Figure 1. The data used was obtained from a previous case-control study (AL-Eitan et al., 2017). Collected data encompassed factors associated with BC risk (ages at BC diagnosis, first pregnancy, menarche, and menopause, allergy, body mass index, breastfeeding status, co-morbidity, family history, and smoking) and prognosis (axillary lymph node status, estrogen, progesterone, and HER2 receptor status, histological classification, molecular subtypes and tumor differentiation, size and stage). In addition, blood samples were withdrawn from the cases and controls for subsequent DNA extraction. 

*DNA extraction and Genotyping*

DNA was extracted from the blood samples using the Wizard^®^ Genomic DNA Purification Kit (Promega Corp., USA) for subsequent quantification and qualification on the Nano-Drop ND-1000 UV-Vis Spectrophotometer (BioDrop, UK). Diluted samples with final concentrations of 20 ng/μl were then shipped on wet ice to the Australian Genome Research Facility (AGRF) in Melbourne for genotyping on the SequenomMassARRAY^®^system (iPLEX GOLD) (Sequenom, USA).

*Statistical analysis*

All investigated SNPs in the cases and controls were tested to fulfill the Hardy-Weinberg equilibrium (Rodriguez et al., 2009). Variations between cases and controls were calculated by employing Pearson’s chi-squared using the Statistical Package for the Social Sciences (SPSS), version 25.0 (SPSS, Inc., Chicago, IL). SNPStats software was utilized for the haplotypic analysis in addition to different genetic model analysis (Sole et al., 2006). The odds ratio (OR) was also calculated using binary logistic regression with 95% confidence intervals (CI). A p-value of 0.05 or lower is considered statistically significant. 

*Correction for Multiple Testing*

However, when multiple comparison tests apply according to the method published by (Li and Ji, 2005) to estimate the effective number of SNPs (Nem) that employs a modification of an earlier approach by Nyholt (2004) . Modified Bonferroni procedure was applied to determine a target alpha level (0.05/ Nem) that would maintain an overall significance level of 0.05 or less.

## Results

*Samples characteristics*

In this study, the general characteristics for controls were summarized and categorized in previously published study by (AL-Eitan et al., 2017). Unrelated healthy females were randomly selected from the Jordanian population with an average age of 50.8 ± 12.6 years.Data obtained for this study were available for 219 female patients who were diagnosed with BC. The averages ages of participantsat BC diagnosis (51.1± 16.5), at pregnancy (22.6± 2), age at menarche (13.8±0 ) and age at menu pause (48.31±4.5). The average body mass index (BMI) was 31.28±3.48for the patient group. Besides, Table 1 describes the clinical and pathological features of BC patients in this study. Three Molecular subtypes of BC depending on estrogen, progesterone, and HER2 status were investigated in this study: luminal A (L. A): ER(+) and /or PR(+) Her2 (-), luminal B (L.B): ER(+) and /or PR(+) Her2 (+), triple-negative (T.N): ER(-) and /or PR(- ) Her2 (-) (Sadek et al., 2017). We found that 47% of patients were L.A, 41% were L.B while 12% were T.N. 

*Genes and their minor allelic frequencies*

Table 2 depicts the candidate genes and their associated polymorphisms. It also illustrates the distribution of the minor allele of each SNP in the cases and controls along with the Hardy-Weinberg equilibrium p-values. All the investigated SNPs were in accordance with HWEexcept for* rs599774* of the* LSP1* gene, which was excluded. 

*Association of investigated SNPs with breast cancer (BC)*

In this study, the correlation between BC and the candidate polymorphisms were investigated. The FGFR2 rs1219648 SNP was associated with BC (p-value = 0.041), but no such association was found for the other studied SNPs, nor were there any significant differences in the allelic and genotypic frequencies between the cases and controls as shown in Table 3. Different genetic models were then incorporated into the analysis to further ascertain the extent of association, and only the CASP8 rs6760993 SNP was found to be significantly associated with BC (p-value = 0.04) upon employing the dominant model are shown in Table 4.

*Association of investigated SNPs with breast cancer (BC) risk and prognosis*

In addition to direct association with the disease, the SNPs included in this study were tested for association with the factors involved in BC risk and prognosis (Tables 5 and 6). The CASP8 rs6760993 SNP was significantly associated with age at menarche (p-value = 0.001). Smoking was significantly associated with the FGFR2 SNP rs1219648 (p-value = 0.01) and the FN1 SNP rs10207245 (p-value = 0.01), and age at menopause was linked to the FGFR2 SNP rs1219648 (p-value = 0.02). Also, the IGF1 SNP rs2373721 was associated with family history of BC (p-value = 0.01). With regard to the prognostic factors, the FGFR2 SNP rs1219648 was associated with tumor differentiation (p-value = 0.018), the MAP3K1 SNP rs889312 was linked to the HER2 marker (p-value = 0.04), and the IGF1 SNP rs2373721 was correlated with progesterone receptor status (p-value = 0.04). 

Additionally, estrogen receptor status was significantly associated with both the LSP1 SNP rs661348 (p-value = 0.02) and the MMP7 SNP rs1943779 (p-value = 0.01).

*Haplotype analysis*

Haplotypes involving two FGFR2 loci are summarized in Table 7. A significant difference in the frequency of the FGFR2 GA haplotype was found between cases and controls (p-value = 0.04), suggesting a reduction in BC risk.

**Table 1:** Clinical and Pathological Features of BC Patients

Clinical Characteristics	Frequency (n=219)	Pathological Characteristics		Frequency (n=219)
BMI				
<=25	24.00%	Progesterone Receptor Status	Positive	44%
>25	76.00%		Negative	55%
First Pregnancy (Age)				
<20	19.00%	Estrogen Receptor	Positive	74.00%
>=20	81.00%		Negative	26.00%
Age at Breast Cancer Diagnosis				
<45	34.00%	Tumor Differentiation	Low Differentiation	38.00%
>=45	66.00%		Mid & High. Differentiation	62.00%
Age at First Menstruation				
<13	30.00%	Axillary Lymph Nodes	Free of tumor	49.00%
>=13	70.00%		Show Metastatic Carcinoma	51.00%
Breastfeeding Status				
Yes	66.30%	Tumor Stage	PT1-PT2	90.00%
No	33.70%		PT3-PT4	10.00%
Age at Menopause				
<50	46.70%	Histology Classification	In Situ Carcinoma	20.00%
>=50	53.30%		Invasive Carcinoma	80.00%
Family History				
Yes	32.00%	Tumor Size (CM3)	<=2	20.00%
No	68.00%		x>2	80.00%
Allergy				
Yes	27.00%	Lymph Node Involvement	Yes	82.00%
No	73.00%		No	18.00%
Smoking				
Yes	30.00%	Human Epidermal Growth Factor Receptor 2 (Her2) Marker	Positive	30.00%
No	70.00%			
Co-morbidity				
Yes	46.00%			
No	54.00%		Negative	60.00%

**Table 2:** Minor Allele Frequencies and Hardy-Weinberg *P*-values of Candidate Gene SNPs

Gene	SNP ID	Cases (n = 221)			Controls (n = 218)		
		MA^a^	MAF^b^	HWE^c ^*P-*Value	MA^a^	MAF^b^	HWE^c ^*P-*Value
*ATM*	rs1800889	T	N/A	NA	T	0.01	NA
	rs1801516	A	0.08	0.18	A	0.08	NA
*CASP8*	rs6760993	A	0.39	0.2	A	0.35	0.17
*FGFR2*	rs1219648	G	0.49	NA	G	0.42	0.58
	rs2981582	A	0.43	0.49	A	0.5	0.79
*FN1*	rs10207245	A	0.32	0.12	A	0.32	0.88
*IGF1*	rs2373721	G	0.29	0.41	G	0.31	0.15
*LSP1*	rs599774	G	0.37	0.01	G	0.33	0.02
	rs661348	C	0.34	0.37	C	0.32	0.76
*MAP3K1*	rs889312	C	0.37	0.08	C	0.55	0.34
*MMP7*	rs1943779	C	0.37	0.77	C	0.34	0.88
*RHOC*	rs2999156	G	0.40	0.89	G	0.44	0.22

^a^MA, Minor Allele; ^b^MAF, Minor Allele Frequency; ^c^HWE, Hardy-Weinberg Equilibrium; NA, Not Applicable.

**Table 3:** Association of the Candidate Gene *SNPs* with Breast Cancer (BC)

Gene	SNP ID	Allelic and Genotypic Frequencies in Cases and Controls				
		Allele/Genotype	Cases (n = 221)	Controls (n = 218)	P-value	Chi-square
*ATM*	rs1800889	C	442 (100)	433(99)	0.09	2.91
		T	0 (-)	3 (01)		
		CC	221 (100)	215 (99)	0.08	3.06
		CT	0 (-)	3 (0.01)		
	rs1801516	G	405 (92)	402 (92)	0.755	0.09
		A	37 (08)	34 (08)		
		G/G	187 (84.6)	185 (84.9)	0.60	1.01
		A/G	31 (14)	32 (14.7)		
		A/A	3 (1.4)	1 (0.5)		
*CASP8*	rs6760993	G	268 (61)	277 (65)	0.20	1.59
		A	170 (39)	147 (35)		
		G/G	77 (35.2)	95 (44.8)	0.06	5.46
		A/G	114 (52)	87 (41)		
		A/A	28 (12.8)	30 (14.2)		
*FGFR2*	rs1219648	A	223 (0.51)	252 (0.58)	0.04	4.17
		G	215 (0.49)	184 (0.42)		
		A/A	57 (26)	75 (34.4)	0.12	4.21
		A/G	109 (49.8)	102 (46.8)		
		G/G	53 (24.2)	41 (18.8)		
	rs2981582	G	219 (50)	248 (57)	0.06	3.60
		A	215 (50)	188 (43)		
		G/G	54 (24.9)	73 (33.5)	0.13	4.07
		G/A	111 (51.1)	102 (46.8)		
		A/A	52 (24)	43 (19.7)		
*FN1*	rs10207245	T	296 (68)	293 (68)	0.86	0.03
		A	142 (32)	137 (32)		
		T/T	105 (48)	99 (46)	0.45	1.58
		T/A	86 (39.3)	95 (44.2)		
		A/A	28 (12.8)	21 (9.8)		
*IGF1*	rs2373721	C	313 (0.71)	303 (0.69)	0.59	0.28
		G	127 (0.29)	133 (0.31)		
		C/C	114 (51.8)	110 (50.5)	0.80	0.43
		G/C	85 (38.6)	83 (38.1)		
		G/G	21 (9.6)	25 (11.5)		
*LSP1*	rs661348	T	293 (66)	297 (68)	0.56	0.33
		C	149 (34)	139 (32)		
		T/T	100 (45.2)	102 (46.8)	0.78	0.49
		C/T	93 (42.1)	93 (42.7)		
		C/C	28 (12.7)	23 (10.6)		

*P*-Value <0.05 was considered as significant.

**Table 4:** Incorporating Different Genetic Models to Carry out Genetic Association Analysis between Candidate Gene SNPs and Breast Cancer (BC)

Gene	SNP ID	Model	Genotype	Cases (%)	Controls (%)	OR	P-Value
						(95% CI)	
*ATM*	rs1801516	Dominant	G/G	187 (84.6%)	185 (84.9%)	1	0.94
			A/G-A/A	34 (15.4%)	33 (15.1%)	1.02 (0.61-1.71)	
		Recessive	G/G-A/G	218 (98.6%)	217 (99.5%)	1	0.31
			A/A	3 (1.4%)	1 (0.5%)	2.99 (0.31-28.93)	
*CASP8*	rs6760993	Dominant	G/G	77 (35.2%)	95 (44.8%)	1	0.04
			A/G-A/A	142 (64.8%)	117 (55.2%)	1.50 (1.02-2.21)	
		Recessive	G/G-A/G	191 (87.2%)	182 (85.8%)	1	0.68
			A/A	28 (12.8%)	30 (14.2%)	0.89 (0.51-1.55)	
*FGFR2*	rs1219648	Dominant	A/A	57 (26%)	75 (34.4%)	1	0.05
			A/G-G/G	162 (74%)	143 (65.6%)	1.49 (0.99-2.25	
		Recessive	A/A-A/G	166 (75.8%)	177 (81.2%)	1	0.17
			G/G	53 (24.2%)	41 (18.8%)	1.38 (0.87-2.18)	
	rs2981582	Dominant	G/G	54 (24.9%)	73 (33.5%)	1	0.05
			G/A-A/A	163 (75.1%)	145 (66.5%)	1.52 (1.00-2.31)	
		Recessive	G/G-G/A	165 (76%)	175 (80.3%)	1	0.28
			A/A	52 (24%)	43 (19.7%)	1.28 (0.81-2.02)	
*FN1*	rs10207245	Dominant	T/T	105 (48%)	99 (46%)	1	0.69
			T/A-A/A	114 (52%)	116 (54%)	0.93 (0.64-1.35)	
		Recessive	T/T-T/A	191 (87.2%)	194 (90.2%)	1	0.32
			A/A	28 (12.8%)	21 (9.8%)	1.35 (0.74-2.47)	
*IGF1*	rs2373721	Dominant	C/C	114 (51.8%)	110 (50.5%)	1	0.78
			G/C-G/G	106 (48.2%)	108 (49.5%)	0.95 (0.65-1.38)	
		Recessive	C/C-G/C	199 (90.5%)	193 (88.5%)	1	0.51
			G/G	21 (9.6%)	25 (11.5%)	0.81 (0.44-1.50)	
*LSP1*	rs661348	Dominant	T/T	100 (45.2%)	102 (46.8%)	1	0.75
			C/T-C/C	121 (54.8%)	116 (53.2%)	1.06 (0.73-1.55)	
		Recessive	T/T-C/T	193 (87.3%)	195 (89.5%)	1	0.49
			C/C	28 (12.7%)	23 (10.6%)	1.23 (0.68-2.21)	
*MMP7*	rs1943779	Dominant	T/T	88 (39.8%)	94 (43.3%)	1	0.46
			T/C-C/C	133 (60.2%)	123 (56.7%)	1.16 (0.79-1.69)	
		Recessive	T/T-T/C	189 (85.5%)	191 (88%)	1	0.44
			C/C	32 (14.5%)	26 (12%)	1.24 (0.71-2.17	
*MAP3K1*	rs889312	Dominant	A/A	94 (42.5%)	97 (44.5%)	1	0.68
			C/A-C/C	127 (57.5%)	121 (55.5%)	1.08 (0.74-1.58)	
		Recessive	A/A-C/A	185 (83.7%)	191 (87.6%)	1	0.24
			C/C	36 (16.3%)	27 (12.4%)	1.38 (0.80-2.36)	
*RHOC*	rs2999156	Dominant	C/C	79 (35.9%)	64 (29.4%)	1	0.14
			C/G-G/G	141 (64.1%)	154 (70.6%)	0.74 (0.50-1.11)	
		Recessive	C/C-C/G	184 (83.6%)	181 (83%)	1	0.86
			G/G	36 (16.4%)	37 (17%)	0.96 (0.58-1.58)	

P- Value <0.05 was considered as significant; OR, Odd Ratio; CI, Confidence Interval.

**Table 5:** Association between Candidate SNPs and BC Risk Factors

Risk Factors	*ATM*	*CASP8*	*FGFR2*		*FN1*	*IGF1*	*LSP1*	*MAP3K1*	*MMP7*	*RHOC*
	rs1801516	rs6760993	rs1219648	rs2981582	rs10207245	rs2373721	rs661348	rs889312	rs1943779	rs2999156
Age at BC Diagnosis **	0.12	0.19	0.82	0.79	0.8	0.15	0.32	0.17	0.46	0.31
Age at First Pregnancy **	0.95	NA	0.88	0.08	0.92	0.83	0.2	0.09	0.82	0.08
Age at Menarche **	0.36	0.001	0.08	0.69	0.17	0.74	0.78	0.79	0.38	0.82
Age at Menopause **	0.46	0.86	0.02	0.34	0.34	0.64	0.15	0.45	0.08	0.07
Allergy *	0.31	0.73	0.49	0.51	0.5	0.67	0.6	0.97	0.56	0.35
Body Mass Index **	0.61	0.29	0.54	0.71	0.7	0.8	0.55	0.4	0.8	0.55
Breastfeeding Status *	0.46	0.56	0.57	0.21	0.28	0.84	0.79	0.19	0.51	0.92
Co-morbidity *	0.28	0.2	0.35	0.92	0.54	0.91	0.08	0.67	0.34	0.38
Family History *	0.22	0.14	0.92	0.89	0.4	0.01	0.81	0.37	0.43	0.17
Smoking *	0.58	0.01	0.01	0.01	0.78	0.73	0.77	0.42	0.72	0.9

*, Pearson’s chi-squared test was used to determine genotype-phenotype association; **, Analysis of variance (ANOVA) was used to determine genotype-phenotype association; P-Value <0.05 was considered as significant

**Table 6:** Association between Candidate SNPs and BC Prognostic Factors

Prognostic Factors	*ATM*	*CASP8*	*FGFR2*		*FN1*	*IGF1*	*LSP1*	*MAP3K1*	*MMP7*	*RHOC*
	rs1801516	rs6760993	rs1219648	rs2981582	rs10207245	rs2373721	rs661348	rs889312	rs1943779	rs2999156
Axillary Lymph Nodes Metastatic *	0.23	0.54	0.12	0.44	0.216	0.67	0.18	0.46	0.3	0.97
Estrogen Receptor status * (Positive vs Negative)	0.21	0.89	0.69	0.51	0.67	0.2	0.02	0.5	0.01	0.37
HER2*	0.09	0.29	0.15	0.21	0.274	o.41	0.97	0.04	0.2	0.41
Histology Classification *	0.58	0.73	0.36	0.42	0.41	0.67	0.4	0.79	0.31	0.32
Molecular Subtypes*	0.02	0.36	0.42	0.65	0.45	0.45	0.56	0.15	0.06	0.2
Lymph Node Involvement *	0.66	0.99	0.97	0.74	0.3	0.24	0.18	0.49	0.23	0.43
Progesterone Receptor Status* (Positive vs Negative)	0.28	0.35	0.48	0.52	0.71	0.04	0.73	0.14	0.63	0.63
Tumor Differentiation*	0.15	0.23	0.02	0.11	0.73	0.26	0.86	0.42	0.75	0.48
Tumor Size **	0.34	0.66	0.91	0.87	0.57	0.83	0.38	0.13	0.84	0.2
Tumor Stage *	0.74	0.29	0.15	0.11	0.07	0.76	0.92	0.71	0.62	0.35

*, Pearson’s chi-squared test was used to determine genotype-phenotype association; **, Analysis of variance (ANOVA) was used to determine genotype-phenotype association; P-Value < 0.05 was considered as significant

**Table 7:** Haplotypic Analysis of *FGFR2* Polymorphisms

Haplotype	Frequency of Block	Frequency Ratio (Case : Control) (%)	Odds Ratio (95% CI)	P-value*
*FGFR2 *Block (rs1219648 and rs2981582)				
AG	0.52	0.4857: 0.5571	1	NA
GA	0.44	0.4741: 0.4103	1.32 (1.01 - 1.73)	0.04
AA	0.02	0.0213: 0.0209	1.19 (0.48 - 2.95)	0.71
GG	0.02	0.0188: 0.0117	1.86 (0.59 - 5.84)	0.29

Global haplotype association p-value: 0.19; *P-Value <0.05 was considered as significant; NA, Not Applicable

## Discussion

 Despite affecting one in five Jordanian women, breast cancer (BC) has been the subject of a relatively limited number of studies in Jordan (AL-Eitan et al., 2019; AL-Eitan et al., 2019; AL-Eitan et al., 2019; AL-Eitan et al., 2019; AL-Eitan et al., 2019; AL-Eitan et al., 2019, AL-Eitan et al., 2019). Protective factors against BC among Jordanian women involved physical activity as well as frequent fruit and vegetable intake, while calcium intake of more than three times a week and postmenopausal obesity were associated with increased BC risk (Al Qadire et al., 2018; Atoum and Al-Hourani, 2004). In terms of genetic association studies, BC risk in Jordanians was linked to mutations in the *BRCA1, DAPK1, MMP9, MTHFR, TP53, *and *TOX3* genes (Atoum and Al-Kayed, 2004; Awwad et al., 2015). The aim of the present study was to determine the extent of the relationship between SNPs in the *ATM, CASP8, FGFR2, IGF1, LSP1, MAP3K, MMP7*, and *RHOC* genes and BC risk and prognosis. To the best of the authors’ knowledge, no other study has attempted to understand such interplay between genetic and environmental factors in BC in the Jordanian population. The findings of the present study indicate that the* rs1219648 SNP* of the *FGFR2* gene is significantly but slightly associated with BC in Jordanian women (p-value = 0.04). Hormones related risk factors such as age at first menstruation, age at menopause and age at first pregnancy may influence the development of breast cancer disease among females, because of the exposure time to endogenous estrogen. In addition, these factors may increase the risk among patients with mutant allele/ genotype.

*rs1219648* was found to be significantly associated with age at menopause (p-value = 0.02), smoking (p-value = 0.01), and tumor differentiation (p-value = 0.02). Lifestyle habit such as smoking is considered as a cancer risk factor, we hypothesized that smoker patients with BC may be at increased risk when carrying mutant alleles. rs1219648 of FGFR2 was significantly associated with smoking status; we found that 39% of smoking cases were carrying AA genotype, while 19% of the nonsmoking cohorts were with the same genotype. Furthermore, 13% of smoking cases were carrying the GG genotype compared to 29% of non-smoking cases with the same genotype. In this regard, we hypothesize that AA genotype may increase the risk of developing BC among women. Correspondingly, the *FGFR2 SNP rs1219648* was associated with BC risk in the various populations, including African-Americans, Asians, and Caucasian-Americans (Rebbeck et al., 2008; Anderson et al., 2006; Zhang et al., 2017). Variation of the associations among different populations may be related to false-positive results due to population stratification. Environmental effects also contribute to genetic variation among the population. However, in this study, all subjects were genetically homogenous (native Arab ancestry).Haplotypic analysis indicated that the FGFR2 GA haplotype conferred a protective effect by reducing BC risk in Jordanian women. Different FGFR2 haplotypes such as GTGT in African-Americans and GTG in north Indians have been reported to increase BC risk (Siddiqui et al., 2014; Barnholtz-Sloan et al., 2010).

None of the other investigated SNPs showed any association with BC in Jordanian patients, but, upon the application of the dominant model (homozygote dominant GG vs heterozygote GA + recessive homozygote AA), the *rs6760993 SNP* of the *CASP8* gene showed association with the disease (p-value = 0.04).This finding reveal an involvement of the dominant genotype GG of *CASP8 *gene variant *rs6760993* with *BC risk. rs6760993* was also linked to ages at menarche (p-value = 0.001) and smoking (p-value = 0.01). No previously published reports were found regarding the role of the CASP8 SNP rs6760993 in the context of BC suggesting that it is not pathogenic. A few SNPs other than rs6760993 in CASP8 are previously associated with subtype-specific breast cancer risk (Park et al., 2016). With regard to the remainder of SNPs, the FN1 SNP rs10207245 was associated with smoking (p-value = 0.01) while the IGF1 SNP rs2373721 was correlated with family history of BC (p-value = 0.01) and progesterone receptor status (p-value = 0.04). Both SNPs were not reported in the context of BC in previous studies. In contrast, the MAP3K1 SNP rs889312, which was linked to HER2 marker status (p-value = 0.04) in our study, was associated with increased BC risk in previously published literature (Rebbeck et al., 2008; Garcia-Closas and Chanock, 2008). Furthermore, our findings show that the LSP1 rs661348 and MMP7 rs1943779 SNPs were both significantly associated with estrogen receptor status. The C allele of the MMP7 rs1943779 SNP was previously shown to have a protective effect on distant metastasis development, but no such report was found for the LSP1 rs661348 SNP (Tapper et al., 2008).

It is apparent that BC etiology in Jordanian women is influenced by different genetic factors compared to those in other populations. While all of the genes included in this study had been previously shown to have a role in BC in other populations, only a select few exhibited a significant association with BC or its risk and prognosis in Jordanians. One limitation of this study is its relatively small sample size, although that can be offset by the fact that this is the first study to investigate these genes in the context of BC as it occurs in Jordan. 
